# Effects of Supplementation of Branches and Leaves Trimmed from Tea Plant on Growth Performance, Rumen Fermentation and Meat Composition of Nanjiang Yellow Goats

**DOI:** 10.3390/ani9090590

**Published:** 2019-08-21

**Authors:** Ali Mujtaba Shah, Yimin Cai, Huawei Zou, Xiangfei Zhang, Lizhi Wang, Bai Xue, Peiqiang Yu, Zhisheng Wang, Quanhui Peng

**Affiliations:** 1 Institute of Animal Nutrition, Key Laboratory of Bovine Low Carbon Farming and Safe Production, Sichuan Agricultural University, Chengdu, Sichuan 611130, China; 2 Department of Livestock Production, Shaheed Benazir Bhutto University of Veterinary and Animal Sciences, Sakrand 67210, Sindh, Pakistan; 3 Japan International Research Center for Agricultural Sciences and Japan National Institute of Livestock and Grassland Science, Tsukuba, Ibaraki 3004352, Japan; 4 Department of Animal and Poultry Science, University of Saskatchewan, Saskatoon, SK S7N 5A8, Canada

**Keywords:** goat, tea plant, antioxidative capacity, meat composition

## Abstract

**Simple Summary:**

The effects of supplementation of branches and leaves trimmed from tea tree (BLTT) on growth performance, rumen fermentation characteristics and meat composition of fattening Nanjiang Yellow goats were studied. Supplementation of 4% BLTT increased final body weight of goats and also increased the activity of superoxide dismutase, while glutathione peroxidase and malondialdehyde followed the quadratic curve. Quadratic curves were also observed for villus height, crypt depth and the ratio of villus height to crypt depth in the jejunum. The quadratic effect was obtained for total essential amino acids, and individual amino acids threonine and leucine. Supplementation of 4% BLTT decreased the saturated fatty acid C16:0, and a quadratic effect was observed for polyunsaturated fatty acid C18:3 (n−3). From the present study, it is concluded that BLTT supplementation has a positive effect on body antioxidative status, gastrointestinal development, rumen fermentation characteristics and overall hence growth performance and meat composition in goats.

**Abstract:**

Thirty-two 6-month-old, healthy Nanjiang Yellow goats (34.6 ± 4.16 kg) were randomly divided into four treatments to evaluate the supplementary effects of branches and leaves trimmed from tea tree (BLTT) on growth performance, rumen fermentation characteristics, and meat composition in fattening goats. The control goats were fed a basal diet. Treatments 1, 2 and 3 were fed 2%, 4% and 6% of BLTT respectively. After a 60 d feeding trial, blood samples were collected for the analysis of the antioxidant profile and goats were slaughtered to obtain the rumen fluid and carcass samples for determination of rumen fermentation characteristics and meat composition perameters. Increased final body weight was observed in goats fed 4% BLTT compared with control. The activity of superoxide dismutase was increased in the 4% BLTT-treated group, while glutathione peroxidase and malondialdehyde followed the quadratic curve. Quadratic curves were also observed for villus height, crypt depth and the ratio of villus height to crypt depth in the jejunum. The quadratic effect was obtained for total essential amino acids, and individual amino acids threonine and leucine. The saturated fatty acid C16:0 was decreased with 4% of BLTT supplementation, and a quadratic effect was observed for polyunsaturated fatty acid C18:3 (n−3). To sum up, our findings revealed that BLTT supplementation has a positive effect on body antioxidative status, gastrointestinal development, rumen fermentation characteristics and overall growth performance and meat quality in goats.

## 1. Introduction

Goats have been raised for fulfilling the meat requirement of human beings for more than 10,000 years. People of pastoral nomadic systems domesticated the goats for dual purpose, i.e., for obtaining both milk and meat [1]. The domestication of goats flourished in tropical and sub-tropical areas, typically in marginal lands, because goats can intake roughage and less concentrate but with high feed-conversion efficiency. Compared to pork or chicken, goat meat is richer in protein, vitamins and has less cholesterol content [2], with rapid economic development, goat meat consumption is also increasing in China year by year.

In Southeast Asian and eastern countries, the consumption of green tea in bottles, packs and cans is vast and increasing. Furthermore, ready-made tea drinks are manufactured by the beverage companies, and the resultant massive amount of byproducts of tea leaves poses a significant problem [3]. Of note, in China, tea trees are widely planted to increase the income of local people in mountainous areas where it is unsuitable for other crops.

It has been reported that tea leaves have the potential to be used as animal feed primarily because of their higher crude protein (21%–28%) content [4,5], especially for animals utilising the low-quality forage in protein-deficient areas. Feed efficiency and body weight gain improvement with the addition of bioactive compounds of green tea in pigs [6], broilers [7], calves [8] and also goats when tea byproducts were included in the diet [3,9]. Furthermore, green tea polyphenols improve growth performance and meat quality parameters, including meat colour, tenderness and shelf life in cattle, sheep and goats [10,11,12]. In a recent study, Brogna et al. [13] reported that feeding of green tea bioactive compounds increased the concentration of C14:1 cis-9 and decreased the cholesterol concentration in sheep meat. However, whether tea trees containing polyphenols could improve goat meat quality by increasing blood antioxidant levels remains unclear. On the other hand, the impact of pruned leaves and branches of tea trees as feed ingredients on gastrointestinal tract development also needs to be investigated. Therefore, this experiment was conducted to determine the effect of branches leaves trimmed tea (BLTT) supplementation on growth performance, antioxidant capacity, gastrointestinal development, rumen fermentation characteristics and meat composition of finishing goats.

## 2. Materials and Methods

All the experimental goats were handled according to the rules of animal care guidelines of Sichuan Agricultural University, China.

Animal Experimentation permission code SYXK (chuan) 2014-184.

### 2.1. Animals and Diets

In this study, 32 Nanjiang Yellow goats (six months old) with an average body weight 34.6 ± 4.16 kg were used and maintained at the animal experimental farm of Sichuan Agricultural University, China. Goats were randomly distributed into four groups (n = 8), i.e., control (fed with a basal diet without BLTT), Treatment 1 (2% BLTT), Treatment 2 (4% BLTT), and Treatment 3 (6% BLTT). The goats were maintained in an intensive housing system (temperature ranges 10–21 °C, humidity 60%–70%) for 60 d formal feeding trial in individual pens (3 m^2^) with elevated floor and ad libitum water access.

The BLTT were obtained from the local area of Sichuan Agricultural University, the ingredients and chemical composition of total mixed ration (TMR) of four experimental diets and BLTT are presented in Table 1A,B, respectively. The diet composition of all of the 4 groups and BLTT were determined in the laboratory of ruminant nutrition.

### 2.2. Proximate Analyses of Total Mixed Ration (TMR) and Branches and Leaves Trimmed from Tea Tree (BLTT)

The BLTT were obtained from a local area of Sichuan Agricultural University China, on 10 May 2017–2018 (after the spring season), which was trimmed by a portable tea pruning machine. After three days of collection, fresh BLTTs were ground through a 3 mm screen for total mixed ration (TMR) formulation. Following stirring, tap water was added to ensure an approximate 40% moisture levels in TMR. Samples were analyzed according to the Association of Official Analytical Chemists (AOAC, 1990) following the method of the Cornell Net Carbohydrate and Protein System (CNCPS). The dry matter (DM) of BLTT and each TMR was determined after drying of samples for 3 h at 135 °C (method 930.15), while the ash content was determined following sample combustion for 6 h at 550 °C (method 942.05). The determination of crude protein (CP) level (CP, 6.25 × N) was achieved using the Kjeldahl method (method 984.13). The phosphorus (P), calcium (Ca), and ether extract, were analysed as per the principles of methods (P; method-965.17), (Ca; method-984.27) and (EE; method 960.39), respectively. The neutral detergent fiber (NDF) was determined (method 930.15) using heat stable amylase according to the procedure of Van Soest et al. [14], acid detergent fiber (ADF; method 973.18), and acid detergent lignin (ADL, method 973.18) contents were determined by an Ankom fiber analyzer described previously by Van Soest et al. [14]. Total phenolics and tannins were determined by the method of Makkar et al. [15] and Makkar and Goodchild [16]. The catechin components of BLTT were analysed using the method described by Ikeda et al. [17] and were determined by high-performance liquid chromatography (HPLC). About 200 mg (±1 mg) of ground tea powders are weighed, added to the centrifuge tube of 20 mL capacity to which 80% aqueous methanol was added and the contents are mixed overnight using an automatic mixer (Karl Hecht ‘Assistant 348′, Sondheim vor der Rhön, Germany) in the dark. After this mixing, all extracts were centrifuged at 4 °C in a refrigerated centrifuge (Baird and Tatlock Ltd., Glasgow, UK) at 3000× *g* for 10 min and each supernatant is transferred into a screwcap brown vial and stored at −20 °C before performing the HPLC analysis. HPLC analysis was undertaken with a HPLC system (Shimadzu, Kyoto, Japan) with auto sampler (SIL-20AC), liquid chromatogram (LC-20AD), degasser (DGU-20AD), column oven (CTO-20AC), photo diode array detector (SPD-M20A) and communication bus module (SBM-20A) was connected to the Shimadzu LC Solution software. A C18 reverse phase column (Spherisorb ODS2, 5 μm, 250 × 4.6 mm i.e., 5 μm, Phenomenex, Cheshire, England) fitted with a guard column (10 mm × 4.6 mm) of the same pace material was used with the column oven set at 40 °C. The eluate ultraviolet (UV) spectra were recorded from 227–550 nm but 270 nm was chosen as the optimum wavelength to identify all the peaks. Two mobile phases, (A) 1% w/v orthophosphoric acid and (B) acetonitrile (≥99.9%), were utilized for gradient elution at 1 mL min using the gradient profile described by Turkmen and Veliooglu [18] as follows: 8% B for 10 min increasing to 18% B at 57 min; 24% B at 78 min; 26% B at 80 min; 28% B at 92 min; 80% B at 98 min; 8% B at 108 min. Column equilibration was undertaken in about 20 min and an automatic batch run started and operated by the Shimadzu LC Solution software integrated to a computer where the injection volume was 20 μL. Each compound was identified and quantified according to the retention time and spectrum view of the corresponding standard. Lipid and water-soluble vitamins were determined by HPLC (Agilent model 1200) as reported by Ersoy & Özeren [19].

### 2.3. Growth Performance

Before formal feeding trial, goats were allowed a 15 d adaptation period for adjusting to the housing and experimental diet plans, and the initial body weight of each goat was recorded on the last day of the adaptation period. The experimental diet was provided twice at 8 a.m. and 6 p.m. The final body weight of each goat was measured on the last day morning of the formal feeding trial before feeding at about 7 a.m. Average daily feed intake (ADFI) was measured as the difference between the amount of feed provided and feed rejection, while the average daily gain (ADG) was calculated as body weight gain (final BW-initial BW) divided by the number of experimental days. The feed conversion ratio was calculated as ADFI/ADG.

### 2.4. Blood Sampling and Parameters Analyses

On the last day of the feeding trial, blood sample was collected from the jugular vein of goats using an 18-G needle (Becton Dickinson Medical(s) Pvt Limited, Singapore). Aliquots of blood samples were immediately put in vacutainer tubes 10 mL containing ethylene diamine tetraacetic acid as an anticoagulant. Then, the blood samples were centrifuged for 15 min at 4 °C at 3000× *g*, and plasma was carefully stored at −20 °C till further use. The concentration of glutathione peroxidase (GSH-Px), superoxide dismutase (SOD) and malondialdehyde (MDA) were determined using the standard commercial kits ((GSH-PX, A005; SOD, A001-1; MDA, A003-1) Jiancheng Bioengineering Institute of Nanjing, Nanjing, China) according to the manufacturer’s instructions.

### 2.5. Small Intestine Development

After the feeding trial the goats were humanely slaughtered, and for gastrointestinal morphology analysis, the jejunum and ileum were collected. A segment (1 cm) from the center between the Meckel’s diverticulum (jejunum) and bile duct entry, and from the distal end of the lower ileum was obtained and fixed in 10% formalin buffered solution (FBS) for 72 h. Each specimen was then embedded in paraffin, and 5 μm sections were cut and stained with eosin and hematoxylin for light microscope examination (Eclipse E600, Nikon Corp., Tokyo, Japan). The following histo-morphological observations were made; villus height, crypt depth and the ratio of villus height to crypt depth in jejunum and ileum. The villus height was measured from the upper part of the villus to the upper part of lamina propria, while the crypt depth was measured from the base upwards to the section of transition between the villus and crypt [20]. We used Image Pro+ v 4.5 software Package (Media Cybernetics, Silver Spring, Washington, USA) for obtaining the morphological measurements.

### 2.6. Rumen Fermentation Characteristics

The ruminal liquid was collected after slaughter in the early morning on the last day of the experiment. The pH meter (D87 HRB, Tokyo, Japan) was used to measure the ruminal fluid pH, and 4 layers gauze was used to separate the ruminal fluid from the feed particles, and centrifuged for 15 min at 1200× *g*. For de-protein, the perchlorate liquid sample was used, and liquid samples were then stored at −20 °C till further use. Potassium hydroxide was used to neutralize the fluid and centrifuged for 10 min at 400× *g* for the deterimination of NH_3_-N and microbial protein (MCP), and for the deterimination of volatile fatty acid (VFA) analyses another perchlorate liquid sample was used. VFA analysis was performed using an HPLC organic acid analysis system (Shimadzu, Kyoto, Japan) The supernatant was shaken with cation exchange resin (Amberlite, IR 120B H AG, ORGANO CORPORATION, Tokyo, Japan) and centrifuged at 6500× *g* for 5 min. The supernatant was passed through a 0.45 µm filter under pressure, and the filtrate was then injected into an HPLC system. The analytical conditions were as follows: column, SCR-101H (7.9 mm × 30 cm) attached to a guard column SCR(H) (4.0 mm × 5 cm) (Shimadzu); oven temperature, 40 °C; mobile phase, 4 mM p-toluenesulfonic acid aqueous solution; reaction phase, 16 mM Bis-Tris aqueous solution containing 4 mM p-toluenesulfonic acid and 100 µM ethylenediaminetetra-acetic acid; flow rate of the mobile and reaction phase, 0.8 mL/1min; detector, conductivity detector (CDD-6A, Shimadzu). Ruminal ammonia nitrogen (NH_3_-N) was measured as described previously by Chaney et al. [21] and, microbial protein (MCP) was determined according to the procedure describe by Makkar et al. [22].

### 2.7. Meat Composition Analysis

After 24 h of storage at 4 °C, carcasses were divided longitudinally into two parts to achieve left and right sides. About 100 g of *Longissimus thoracis* (LM) was cut from the left half of each carcass for parameters determination. Chemical composition (ash, crude protein and ether extract content) analysis was determined by proximate analysis as described by Lukač et al., (2015) [23]. Free amino acid (AA) concentrations were determined according to the method of Iwaki et al. [24] using cation exchange chromatography and lithium citrate buffer solutions (LKB Biochrom amino acid analyzer, model 4151 Alpha Plus, Pharmacia, Uppsala, Sweden). Fatty acids content was determined by gas chromatography after lipid saponification and fatty acids esterification [25] after fat saponification and esterification with 13%–15% BF3/methanol. Heptadecanoic acid was used as a reference. A gas chromatograph Varian CP-3800 equipped with a capillary column (CP WAX 52CB, 60 m × 0.25 mm × 0.25 µm; Varian) and a FID detector was used. The injector and detector were maintained at 260 °C, and oven temperature was 200. 1 µL sample was injected with helium as the carrier gas at 1.4 mL/min, and split of 1:10. The fatty acids were divided into saturated fatty acids (SFA), monounsaturated fatty acids (MUFA), and polyunsaturated fatty acids (PUFA).

### 2.8. Statistical Analyses

Data were subjected to analysis of variance (ANOVA) by the PROC MIXED MODEL of the Statistical Analysis System (SAS In., Cary, USA). Individual goats served as the random experimental units for all of the data, and the BLTT served as the fixed effect. Orthogonal polynomials were used to calculate the linear and quadratic effect of the BLTT in diets on parameters, and for comparision of means the Tukey-Kramer method was used. The results were presented as the least-squares means with the standard error of the mean (SEM). Significance was described as *p* ≤ 0.05, while 0.5 < *p* < 0.10 was regarded as a trend.

## 3. Results

### 3.1. Growth Performance

The results of the growth performance of goats are presented in Table 2. Supplementation of BLTT showed a quadratic effect (*p* = 0.039) on the final body weight of goats, and a quadratic trend (*p* = 0.070) was also observed in ADG. However, no significant effect was observed on the ADFI and feed conversion ratio (FCR) among the four groups.

### 3.2. Antioxidant Capacity

The results of the antioxidant capacity are presented in Table 3. Briefly, supplementation of 4% BLTT increased (*p* = 0.001) plasma SOD activity. In addition, both GSH-Px and MDA followed quadratic curve (*p* < 0.001) following BLTT supplementation.

### 3.3. Small Intestine Development

**Figure 1:**
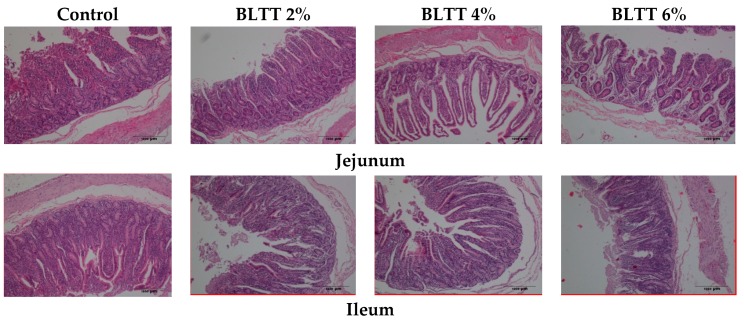
Histological diagram of sections of small intestine (hematoxylin and eosin staining) in each group. BLTT = Branches leaves trimmed tea plant.

The results of the small intestine development of goats are presented in Table 4 and Figure 1. Briefly, supplementation of BLTT quadratically affected the villus height (*p* = 0.029), crypt depth (*p* = 0.001) and villus height: crypt depth (*p* = 0.001) in the jejunum. Supplementation of 2% BLTT also increased the villus height of ileum (*p* = 0.005). Furthermore, a linear trend (*p* = 0.053) was observed in the ratio of villus height to crypt depth in ileum following BLTT supplementation.

### 3.4. Rumen Fermentation Characteristics

The results of rumen fermentation characteristics in goats are presented in Table 5. Supplementation of BLTT showed quadratic effect on the molar proportion of acetate (*p* = 0.002), propionate (*p* < 0.001), MCP (*p* < 0.001), NH_3_-N (*p* = 0.001), and ratio of acetate to propionate (*p* = 0.001). Furthermore, a quadratic trend (*p* = 0.086) was observed for rumen pH following supplementation of BLTT.

### 3.5. Muscle Composition

The results of goat muscle chemical composition are presented in Table 6. Supplementation of BLTT showed quadratic effect on moisture % (*p* = 0.006) and fat % (*p* = 0.019) of LM of goats. However, no significant difference was detected in protein % and ash % between the four groups.

### 3.6. Amino Acids Profile

The results of muscle amino acids profile are presented in Table 7. Supplementation of BLTT showed quadratic effect on total essential amino acids (TEAA) (*p* = 0.046), threonine (*p* = 0.044), and leucine (*p* = 0.009). As for non essential amino acids (NEAA), the glutamine was decreased with 2% BLTT supplementation compared to the control group. Furthermore, a quadratic trend was observed for total non essential amino acids (TNEAA) (*p* = 0.060) with BLTT supplementation.

### 3.7. Fatty Acids Profile

The results of muscle fatty acid are presented in Table 8. The SFA C16:0 was decreased (*p* = 0.037) with 4% BLTT supplementation, and a linear trend was observed for MUFA (*p* = 0.052). A quadratic effect was observed for PUFA C18:3 (*p* = 0.012) with BLTT supplementation.

## 4. Discussion

### 4.1. Growth Performance

Present results showed that dietary BLTT supplementation (4%) increased general weight gain in goats. It is reported that tea by-products are a rich source of crude protein and may have a beneficial influence on feed intake and weight gain in animals [4]. Besides, tea trees possess a high level of essential oils, which act as a growth promoter and are also implicated in modulating the ruminal and intestinal microbiota populations and digestibility of nutrients [26]. Generally, goats prefer plant browsing, and plants contain certain bioactive compounds such as polyphenols. Goats and sheep exposed to secondary compounds of tree plants are highly resistant to gastrointestinal nematodes, which will beneficial for the growth performance of animals [27].

In the present study, a quadratic trend was observed in ADG, and the highest ADG was seen in the 4% supplementation group. In addition, we observed that 6% BLTT supplementation decreased the feed intake compared to the other treated groups; however, it was similar to the control group. This might be attributed to the anti-nutritional factors like alkaloids, which have an adverse influence due to their bitter taste, and ingredients like tannins decrease the digestibility of nutrients [3]. The alkaloids determined in BLTT were 6.89%, and an anti-palatability effect of lupin alkaloids was observed in goats [3]. Interestingly, piperidine alkaloids are acutely toxic to adult livestock species and produce musculoskeletal deformities in neonatal animals [28]. We speculate that inclusion of 6% BLTT might have caused digestive discomfort to the goats; therefore, the feed intake was decreased. Conversely, 4% BLTT supplementation in the diet resulted in improved growth performance, indicating that BLTT may be used as a feed supplement for goats but at appropriate levels with proper inclusion.

### 4.2. Antioxidant Capacity

The quadratic effect was observed in GSH-Px and MDA following supplementation of BLTT. MDA levels were reduced, while the GSH-Px activity was increased. These findings are in line with Yan et al. [29], who reported that green tea catechins feeding decreased the reactive oxygen species (ROS) content in serum, and decreased the MDA content in the serum of mice. Freese et al. [30] reported that green tea reduced plasma MDA in human. Furthermore, Khan et al. [31] reported that when mice were treated with 0.2% w/v of polyphenols extracted from green tea for 30 days, the activity of glutathione peroxidase in pulmonary and hepatic tissues was increased. The antioxidative activity of green tea polyphenols has been intensively studied in the past and regarded as the major mechanisms of action accounting for its health promotional and disease preventive effects. However, some investigators have reported toxicity at higher doses, which is presumably due to the pro-oxidative properties. Murakami et al. [32] showed that diets containing high doses (0.5%–1%) of green tea polyphenols deteriorated dextran sodium sulfate (DSS)-induced intestinal inflammation in rats. These results showed that the beneficial functions of green tea polyphenols act in a dose-dependent manner. These observations corroborate, at least partially, to the quadratic effect observed in growth performance and other parameters in our present study.

### 4.3. Intestinal Development

The BLTT may improve growth performance of the goats through numerous mechanisms, among them, the modification in small intestine histological parameters is an important one, which consequently influences nutrients absorption. In the present study, BLTT supplementation enhanced villus height and the ratio of villus height to crypt depth in the jejunum. It is reported that green tea exerted a protective effect in the intestinal mucosa of mice from fasting-induced or ischemia-reperfusion damage [33,34]. It is believed that an improved villus height is paralleled by a promoted absorptive function of the small intestine because of the larger absorptive surface area [35,36]. The epithelial lining of the intestinal tract is continuously renewed; longer villi are linked with activated cell mitosis [37]. The cellular proliferation in the intestine can be increased or decreased in a wide variety of different circumstances. Insufficient cell production or increased cell loss may result in ulceration or atrophy, and deeper crypts show fast tissue turnover to permit renewal of the villus [38]. Therefore, the increased ratio of villus height to crypt depth in the present study proposing an improved epithelial cell turnover by addition of 4% BLTT, however addition of 2% and 6% BLTT also improved, but not significantly, the ratio of villus height to crypt depth.

### 4.4. Rumen Fermentation Characteristics

The VFAs are considered as one of the rumen fermentation indexes and reflect the rumen fermentation pattern [39]. In the present study, the production of propionate % in BLTT supplementation groups was higher, and the ratio of acetate to propionate was lower than the control, which means that the fermentation pattern was changed. In ruminants, approximate 91 to 95% of propionate produced in the rumen is recovered in the portal vein, of which over 90% is efficiently taken up by the liver [40], and approximately 90% of propionate taken up by the liver is used for glucose production [41,42]. The increase in propionate and decreased ratio of acetate to propionate might imply promoted energy utilization efficiency, especially in the 4% BLTT group.

On the other hand, the concentration of NH_3_-N was reduced, and MCP production was improved by BLTT supplementation. The MCP production was greater in the group of BLTT supplements compared to the control group, and 4% BLTT addition in the diet resulted in the highest MCP production and lowest NH_3_-N concentration. It is reported that the complex polyphenols in the context of a model system can modulate select members of the human gut microbiota [43]. It is also reported that green tea ground silage had an effect on inhibiting the growth of pathogenic bacteria, and improving rumen microbiota in dairy cows [44]. The alteration in rumen characteristic is important for higher nitrogen utilization efficiency.

### 4.5. Meat Composition

The present study showed a decrease in moisture content in LM with 4% BLTT supplementation, which was in line with researches conducted by Hossain et al. [45] who reported decreased moisture content in a muscle when pigs were fed green tea by-products. A quadratic curve was observed in crude fat content in the present study with BLTT supplementation. It is documented that tea catechins affect the lipid catabolism by decreasing the activity of pancreatic lipase and through luminal emulsification, hydrolysis and micellar solubilization of lipids [46], which may be the potential mechanism for its lipid uptake, and subsequently low-fat deposition. This was also mirrored by Ahmed et al. [3], who observed decreased fat content in goat meat after green tea by-products supplementation.

Although the BLTT supplementation increased the individual concentration of the leucine, threonine and serine in LM of goats, proposing that suitable BLTT supplementation change amino acids constituent movement to the small intestine that might result from the increased absorption of these nutrients from the small intestine. Moreover, an increased level of leucine may increase the protein synthesis in skeletal muscles through the activation of the signalling feature of the mammalian target of rapamycin [47]. Chapman et al. [48] pointed out that threonine is a crucial amino acid which is essential in the gut mucins production and plays a vital role in the formation of tooth enamel, elastin and collagen in mammals. In addition, in healthy adults, the serine has been found to improve the executive function and memory [49]. Increased serine was also observed by Zhong et al. [50]. The change of amino acid profiles triggered by BLTT supplementation may exert a beneficial effect on human health.

Nowadays, consumers are progressively conscious of the association that the consumption of some fatty acids is good for cardiovascular health. PUFA has a good effect on the health of humans, while on the health of humans, the SFA has adverse effects [51,52]. The present study’s results showed that appropriate amounts of BLTT linearly decreased the SFA and also MUFA, while they quadratically increased C18:3(n−3), these results are in agreements with Tan et al. [9] who suggested that dietary addition of tea catechins could reduce the SFA and MUFA and also enhanced the content of C18:3(n−3) in meat of goat. It is reported the tea catechins could hamper the peroxidation of PUFA due to an antioxidant capability [12], On the other hand, tea catechins inhibit the activity of rumen microorganisms reducing bio-hydrogenation of feed PUFA. Hence, it is suggested that the dietary addition of BLTT protects PUFA from the diet and deposition in animal tissues. In addition, present study results showed that the antioxidant capacity in blood improved with the supplementation of BLTT and the results are in agreement with the previous study of the Nishida et al. [53] who provided green tea silage to dairy cows and obtained higher levels of plasma antioxidant capacity and improved health conditions in dairy cows. It would appear that the major antioxidant in green tea other than vitamins A and E is catechine. Given that there was a significant increase in the antioxidant capacity in the plasma of the goats after the BLTT supplementation in this study, the catechins could be the possible agent and from the antioxidant capacity in blood we can recognize the health status and growth performance of the goats.

## 5. Conclusions

To sumup, BLTT supplementation improved the antioxidant capacity, rumen fermentation characteristics and gastrointestinal development and overall growth performance in goats. Dietary supplement of BLTT could also improve the meat composition in goats. Therefore, based on the enticing findings of our study, it is reasonable to recommend that 4% of BLTT, on a dry matter basis, may be included in the ration of finishing goats. Nevertheless, future functional studies will add more value to the current understanding of the potential nutritional benefits of the tea plant in general and BLTT in particular.

## Figures and Tables

**Table:** (**A**)

Ingredients	Control	T1	T2	T3
Corn	45.5	45.5	45.5	45.5
Wheat bran	5.0	5.0	5.0	5.0
Soybean meal	3.0	3.0	3.0	3.0
Canola meal	3.0	3.0	3.0	3.0
Sodium bicarbonate	0.4	0.4	0.4	0.4
Salt	0.5	0.5	0.5	0.5
Vitamin and minerals premix ^1^	0.8	0.8	0.8	0.8
Calcium hydrophosphate	0.8	0.8	0.8	0.8
Calcium carbonate	1.0	1.0	1.0	1.0
Rice straw	15.0	14.0	13.0	12.0
Corn silage	15.0	14.0	13.0	12.0
DDGS	10.0	10.0	10.0	10.0
BLTT ^2^	0.0	2.0	4.0	6.0
Nutritive content ^3^				
DM	76.86	76.31	75.76	75.21
DE (MJ/kg) ^4^	11.33	11.34	11.36	11.38
CP	10.89	11.05	11.13	11.32
EE	2.89	3.03	3.01	3.04
NDF	31.17	30.18	29.79	29.14
ADF	18.66	18.34	18.32	18.11
Ash	6.31	6.26	6.21	6.16
Ca	0.82	0.83	0.84	0.88
P	0.40	0.41	0.42	0.43

^1^ The premix provides per kg diet: Fe in the form of sulfate 60 mg, Cu in the form of sulfate 8 mg, Zn in the form of sulfate 40 mg, Mn in the form of sulfate 40 mg, Co in the form of chloride 0.2 mg, I in the form of iodate 0.3 mg, Se in the form of selenite 0.2 mg and, Vitamin A 10000 IU, Vitamin D 1000 IU, and Vitamin E 80 IU; ^2^ BLTT, Branches and leaves trimmed from the tea tree; ^3^ DE of BLTT was calculated with the equation DE (MJ/kg) = 3813.89 − 15.94 * CF (%) − 13.94 * CP (%), and the ^4^ DE of TMR was calculated values based on the formula DE (MJ/kg) available DE data of the ingredients used. CP, EE, NDF, ADF, Ca and P were calculated values according to the formula; DDGS = Distillers Dried Grains with Solubles, DM = dry matter, D = Digestable energy, CP = Crude protein, EE = Ether extract, NDF = Neutral Detergent Fiber, ADF = Acid Detergent Fiber, Ca = Calcium, P = Phosphorus.

**Table:** (**B**)

Content	Quantity
Dry matter (%)	36.62
CP (%)	20.14
Ether Extract (%)	03.10
NDF (%)	35.26
ADF (%)	28.43
ADL (%)	03.53
Ca (%)	0.24
P (%)	0.11
Catechins	(%)
Catechin	0.34
Epicatechin	1.74
Epigallocatechin	5.27
Epicatechin gallate	2.14
Epigallocatechin-3 gallate	8.52
Total Phenolic acid	1.79
Total Alkaloids	6.89
Total Caffeine	1.88
Total Tannins	0.22
Essential Amino acid	mg/100 g
Lysine (Lys)	0.33
Methionine (Met)	0.07
Threonine (Thr)	0.48
Valine (Val)	0.39
Isoleucine (Ile)	0.03
Leucine (Leu)	0.06
Phenylalanine (Phe)	0.18
Vitamins	mg/100 g
Vitamin A	1.12
Vitamin E	22.10
Vitamin B_1_	1.92
Vitamin B_2_	8.12
Vitamin C	21.20
Vitamin K	7.12

**Table 2:** Effects of tea supplementation on growth performance of finishing goats.

Treatments						*p*-Value	
Item	Control	T1	T2	T3	SEM	Linear	Quadratic
Initial BW (kg)	32.4	32.7	32.4	32.3	0.550	0.638	0.748
Final BW (kg)	41.5 ^b^	42.9 ^a,b^	43.7 ^a^	41.5 ^b^	0.454	0.014	0.039
ADG (g)	151.7 ^b^	169.7 ^a,b^	189.9 ^a^	150.7 ^b^	10.275	0.144	0.070
ADFI (g)	1234	1316	1391	1218	44.5	0.111	0.108
FCR	8.82	7.82	7.75	8.43	0.656	0.289	0.471

^a, b^ Means within a row with different superscripts differ (*p* < 0.05); T1, T2 and T3 = supplementation of 2%, 4% and 6% of branches and leaves trimmed from the tea tree. ADG = average daily gain, ADFI = average daily feed intake, FCR = feed conversion ratio; SEM, standard error of the mean.

**Table 3:** Effects of tea supplementation on plasma antioxidant capacity of finishing goats.

Treatments						*p*-Value	
Item	Control	T1	T2	T3	SEM	Linear	Quadratic
SOD (U/mL)	76.77 ^b^	82.12 ^a,b^	86.24 ^a^	75.80 ^b^	1.700	0.001	0.472
GSH-Px (U/mL)	157.55 ^b^	163.64 ^a,b^	169.73 ^a^	160.64 ^a,b^	3.453	0.002	<0.001
MDA (nmol/mL)	5.74 ^a^	4.41 ^b^	4.47 ^b^	5.81 ^a^	0.311	<0.001	<0.001

^a,b^ Means within a row with different superscripts differ (*p* < 0.05); T1, T2 and T3 = supplementation of 2%, 4% and 6% of branches and leaves trimmed from the tea tree. SOD = superoxide dismutase, GSH-Px = glutathione peroxidase, MDA = malondialdehyde; SEM, standard error of the mean.

**Table 4:** Effects of tea supplementation on small intestine development of finishing goats.

Treatments						*p*-Value	
Item	Control	T1	T2	T3	SEM	Linear	Quadratic
Jejunum							
Villus height (μm)	591.3 ^b^	608.5 ^a,b^	637.9 ^a^	587.5 ^b^	10.73	0.153	0.029
Crypt depth (μm)	261.1 ^a,b^	264.9 ^a^	242.6 ^c^	245.9 ^b,c^	4.700	0.172	0.001
Villus height/Crypt depth	2.27 ^b^	2.30 ^b^	2.63 ^a^	2.39 ^a,b^	0.069	0.991	0.001
Ileum							
Villus height (μm)	471.8 ^b^	500.1 ^a^	495.3 ^a,b^	483.1 ^a,b^	6.53	0.005	0.175
Crypt depth (μm)	239.0	240.8	241.1	246.4	4.067	0.898	0.504
Villus height/Crypt depth	1.98	2.08	2.06	1.97	0.044	0.053	0.682

^a–c^ Means within a row with different superscripts differ (*p* < 0.05); T1, T2 and T3 = supplementation of 2%, 4% and 6% of branches and leaves trimmed from the tea tree. SEM, standard error of the mean.

**Table 5:** Effects of tea supplementation on rumen fermentation characteristics of finishing goats.

Item	Treatments				SEM	*p*-Value	
	Control	T1	T2	T3		Linear	Quadratic
pH	5.86	5.89	6.18	6.24	0.173	0.708	0.086
Acetate (%)	63.44 ^a^	57.61 ^b^	58.07 ^b^	58.83 ^b^	1.534	0.025	0.002
Propionate (%)	16.91 ^b^	16.75 ^b^	19.01 ^a^	17.54 ^a,b^	0.034	<0.001	<0.001
Butyrate (%)	5.82	5.98	6.24	5.63	0.450	0.680	0.687
TVFA (mmol/L)	90.05	87.00	89.68	89.20	1.459	0.143	0.694
MCP (mg/dL)	6.60 ^c^	7.21 ^b,c^	8.41 ^a^	7.78 ^a,b^	0.288	0.459	<0.001
NH_3_-N (mg/dL)	29.88 ^a^	27.01 ^a,b^	23.45 ^b^	24.95 ^a,b^	1.292	0.390	0.001
Acetate: Propionate	3.76 ^a^	3.47 ^a,b^	3.06 ^b^	3.37 ^a,b^	0.115	0.214	<0.001

^a–c^ Means within a row with different superscripts differ (*p* < 0.05); T1, T2 and T3 = supplementation of 2%, 4% and 6% of branches and leaves trimmed from the tea tree. SEM = standard error of the mean, TVFA = total volatile fatty acids, MCP = microbial protein, NH_3_-N = ammonia nitrogen.

**Table 6:** Effects of tea supplementation on muscle composition (%) of finishing goats.

Item	Treatments					*p*-Value	
	Control	T1	T2	T3	SEM	Linear	Quadratic
Moisture	71.04 ^a,b^	70.24 ^b,c^	69.34 ^c^	71.38 ^a^	0.375	0.008	0.006
Protein	20.95	21.30	21.97	21.03	0.407	0.494	0.155
Fat	6.69 ^a^	6.41 ^a^	6.08 ^b^	6.37 ^a^	0.156	0.336	0.019
Ash	0.97	1.06	0.97	0.95	0.038	0.061	0.274

^a–c^ Means within a row with different superscripts differ (*p* < 0.05); T1, T2 and T3 = supplementation of 2%, 4% and 6% of branches and leaves trimmed from the tea tree. SEM, standard error of the mean.

**Table 7:** Effects of tea supplementation on muscle amino acids profile (mg/g protein) of finishing goats.

Item	Treatments					*p*-Value	
	Control	T1	T2	T3	SEM	Linear	Quadratic
TEAA	90.69	92.38	93.41	92.07	0.775	0.191	0.046
Lys	15.59	16.43	16.19	15.74	0.386	0.110	0.689
Phe	7.74	8.05	7.81	7.98	0.135	0.188	0.973
Met	5.11	4.88	5.00	5.36	0.157	0.116	0.669
Thr	8.05	7.97	8.40	8.18	0.140	0.525	0.044
Ile	7.58	7.76	7.84	7.97	0.187	0.805	0.247
Leu	14.59 ^b^	15.01 ^a,b^	15.51 ^a^	14.66 ^b^	0.178	0.067	0.009
Val	7.09	7.34	7.29	7.07	0.129	0.111	0.720
Arg	12.88	12.56	13.11	12.85	0.463	0.612	0.574
His	6.06	6.22	6.08	6.06	0.145	0.387	0.774
Cys	1.65	1.66	1.66	1.61	0.060	0.765	0.959
Tyr	4.36	4.50	4.51	4.59	0.085	0.536	0.212
TNEAA	86.45	86.20	87.95	87.32	0.678	0.569	0.060
Asp	15.33	16.37	16.29	16.31	0.382	0.138	0.164
Ser	7.95	7.36	7.62	7.95	0.176	0.011	0.900
Glu	29.54 ^a^	26.56 ^b^	27.22 ^a,b^	28.17 ^a,b^	0.760	0.012	0.247
Gly	8.61	8.98	8.96	8.72	0.192	0.178	0.476
Ala	9.88	9.77	9.88	10.11	0.182	0.419	0.577

^a,b^ Means within a row with different superscripts differ (*p* < 0.05); T1, T2 and T3 = supplementation of 2%, 4% and 6% of branches and leaves trimmed from the tea tree. SEM, standard error of the mean; TEAA, total essential amino acids; TNEAA, total non-essential amino acids.

**Table 8:** Effects of tea supplementation on muscle fatty acids profile (g/100 g sample) of finishing goats.

Item	Treatment					*p*-Value	
	Control	T1	T2	T3	SEM	Linear	Quadratic
Myristic acid C14:0	0.162	0.162	0.161	0.162	0.003	0.849	0.927
Pentadecylic acid C15:0	0.029	0.03	0.028	0.028	0.013	0.483	0.53
Palmitic acid C16:0	2.197 ^a^	2.163 ^a,b^	2.035 ^b^	2.115 ^a,b^	0.04	0.037	0.178
Palmitoleic acid C16:1	1.805	1.751	1.751	1.741	0.031	0.371	0.27
Margaric acid C17:0	0.164	0.163	0.164	0.167	0.003	0.499	0.758
Stearic acid C18:0	1.626	1.677	1.645	1.644	0.033	0.277	0.995
Octadecenoic acids C18:1	2.143	1.978	1.996	2.039	0.049	0.037	0.165
Linoleic acid C18:2 (n−6)	3.62 ^b^	3.82 ^a^	3.66 ^b^	3.66 ^b^	0.066	0.427	0.341
α-Linolenic acid C18:3 (n−3)	0.26 ^b^	0.27 ^b^	0.29 ^a^	0.29 ^a^	0.01	0.937	0.012
Arachidic acid C20:0	0.012	0.012	0.011	0.012	0.0004	0.915	0.136
Eicosatetraenoic acid C20:4 (n−3)	0.011	0.01	0.011	0.011	0.0003	0.092	0.325
Eicosapentaenoic acid C20:5 (n−3)	0.008	0.008	0.008	0.008	0.0006	0.492	0.807
Behenic acid C22:0	0.016	0.017	0.016	0.017	0.0003	0.199	0.129
Docosapentaenoic acid C22:5 (n−30)	0.028	0.024	0.026	0.024	0.001	0.138	0.263
Lignoceric acid C24:0	0.012	0.011	0.011	0.012	0.0003	0.134	0.762
Rumenic acid c9, t11-CLA	0.026	0.026	0.026	0.026	0.0003	0.61	0.983
t10, c1-2CLA	0.017	0.017	0.017	0.017	0.0003	0.882	0.783
∑SFA	4.218	4.235	4.071	4.157	0.046	0.262	0.245
∑MUFA	3.948	3.729	3.749	3.783	0.067	0.052	0.133
∑PUFA	3.970	4.175	4.038	4.036	0.003	0.984	0.741

^a–c^ Means within a row with different superscripts differ (*p* < 0.05); T1, T2 and T3 = supplementation of 2%, 4% and 6% of branches and leaves trimmed from the tea tree. SFA, saturated fatty acid; MUFA, monounsaturated fatty acid; PUFA, polyunsaturated fatty acid; SEM, standard error of the mean.
